# Fidaxomicin as a Salvage Therapy for Fulminant Clostridioides difficile Infection

**DOI:** 10.7759/cureus.16559

**Published:** 2021-07-22

**Authors:** Caio T Heleno, Aleksey Tagintsev, Katharine Lasley, Douglas Summerfield

**Affiliations:** 1 Internal Medicine, MercyOne North Iowa Medical Center, Mason City, USA; 2 Critical Care Medicine, MercyOne North Iowa Medical Center, Mason City, USA

**Keywords:** c. difficile, fidaxomicin, fulminant c. difficile infection, oral vancomycin, metronidazole

## Abstract

Few studies have demonstrated the efficacy of fidaxomicin in the treatment of fulminant *Clostridioides difficile* infection (CDI). Fidaxomicin has been used as part of the standard treatment for nonsevere and severe CDI according to the guidelines by the Infectious Diseases Society of America and the Society for Healthcare Epidemiology of America, but not in severe fulminant CDI due to lack of randomized clinical trials supporting its use. We present the case of a patient who developed severe fulminant colitis initially refractory to treatment with oral vancomycin and intravenous metronidazole that had an impressive improvement within 24-hour of starting fidaxomicin. The patient had a complete resolution of the symptoms at the end of the therapy without requiring a surgical approach. There are few case reports of fulminant CDI treated with fidaxomicin as a salvage therapy for fulminant CDI. In this challenging scenario, information about the use of fidaxomicin is still limited and more clinical trials are needed to support its widespread use.

## Introduction

Formerly *Clostridium*, *Clostridioides difficile* is a gram-positive, anaerobic, toxin-producing bacteria that colonize the human intestinal tract after the normal gut flora has been disrupted. It is the leading cause of hospital-acquired diarrhea with increasing morbidity and mortality with a total incidence of 130.2 per 100,000 patients according to the Centers for Disease Control and Prevention in the year 2017 [1]. *C. difficile* infection (CDI) can cause a large spectrum of manifestations ranging from an asymptomatic carriage to fulminant disease with toxic megacolon symptoms. Its cardinal symptom is diarrhea, usually defined as three or more episodes of loose stools or 200 g output within a 24-hour period, that can be associated with lower abdominal pain and cramping, low-grade fever, nausea, and anorexia in a setting of a recent history of hospitalization and antibiotic use as well as due to advanced age and proton pump inhibitor suppression of gastric acid [2-5].

CDI can be diagnosed by several diagnostic methods, most commonly by nucleic acid amplification testing (NAAT) alone but also in combination with tests using an enzyme immunoassay (EIA) for glutamate dehydrogenase (GDH) and enzyme immunoassay for *C. difficile* toxins A and B. Less commonly, CDI can be confirmed by cell culture cytotoxicity assay and selective anaerobic culture. EIA for GDH has high sensitivity and poor specificity as detection cannot distinguish between toxigenic and nontoxigenic strains, whereas EIA for toxins A and B has poor sensitivity and high specificity. Although NAAT (which includes polymerase chain reaction) can detect one or more genes specific to toxigenic strains, of which *tcdB *is the most important gene, which encodes for toxin B, it cannot test for active toxin protein production and is capable of detecting asymptomatic carriers of *C. difficile* [5].

Management of CDI is determined based on its severity that is categorized into nonsevere, severe, and fulminant colitis infection. The clinical practice guidelines of the Infectious Diseases Society of America (IDSA)/Society for Healthcare Epidemiology of America (SHEA) define severe disease as the clinical criteria for CDI infection associated with white blood cell (WBC) count of >15,000 cells/µL and/or creatinine >1.5 mg/dL. The fulminant disease is defined by the presence of decompensating clinical features such as hypotension, shock, ileus, or megacolon associated with the criteria for severe disease [5]. Either oral vancomycin or fidaxomicin is recommended for the initial episode of nonsevere and severe CDI. However, for fulminant CDI, the recommendation is to use parenteral metronidazole in addition to oral vancomycin due to concerns about the presence of concomitant ileus or associated condition that prevents oral intake or absorption of antibiotics. There is little efficacy data available for fidaxomicin in this setting likely because, in many clinical trials, patients with fulminant CDI had been excluded from the studied population limiting the knowledge of fidaxomicin’s use in critically ill patients [5-7]. Fulminant CDI that has failed to previous use of vancomycin and metronidazole is especially understudied. Surgery is indicated for colonic perforation, necrosis or full-thickness ischemia, intra-abdominal hypertension, abdominal compartment syndrome, megacolon, clinical signs of peritonitis, end-organ failure, or worsening abdominal symptoms despite adequate medical therapy.

The following case reports a patient with fulminant CDI who was admitted to the intensive care unit (ICU) with septic shock and renal insufficiency requiring urgent dialysis. He displayed a fast response to fidaxomicin use with clinical improvement after having failed to improve with parenteral metronidazole and oral vancomycin use.

## Case presentation

Here, we report the case of a 60-year-old Caucasian male patient with a past medical history of diabetes mellitus type 2, coronary artery disease, atrial fibrillation, hypertension, obstructive sleep apnea, chronic kidney disease stage 3, and nonalcoholic steatohepatitis. The patient had a recent hospital admission due to a surgical C2-C3 epidural abscess which was treated with drainage and spinal C2-C5 laminectomy and fusion, initially manifesting as quadriparesis. Abscess cultures were positive for methicillin-sensitive *Staphylococcus aureus*, and he was completing his six-week treatment of intravenous (IV) cefazolin in a skilled nursing facility after hospitalization. Nearly 10 days after hospital discharge, the patient started developing diffuse abdominal pain, decreased appetite, poor oral intake, and diarrhea. He tested positive for *C. difficile* and was started on oral vancomycin. Two days after starting the antibiotic, his clinical status rapidly declined with worsening diarrhea, oliguria, and hypotension. Although he was initially transferred to a regular floor in another hospital, he was admitted to our ICU two days later with septic shock, respiratory failure, and acute kidney injury.

**Figure 1:**
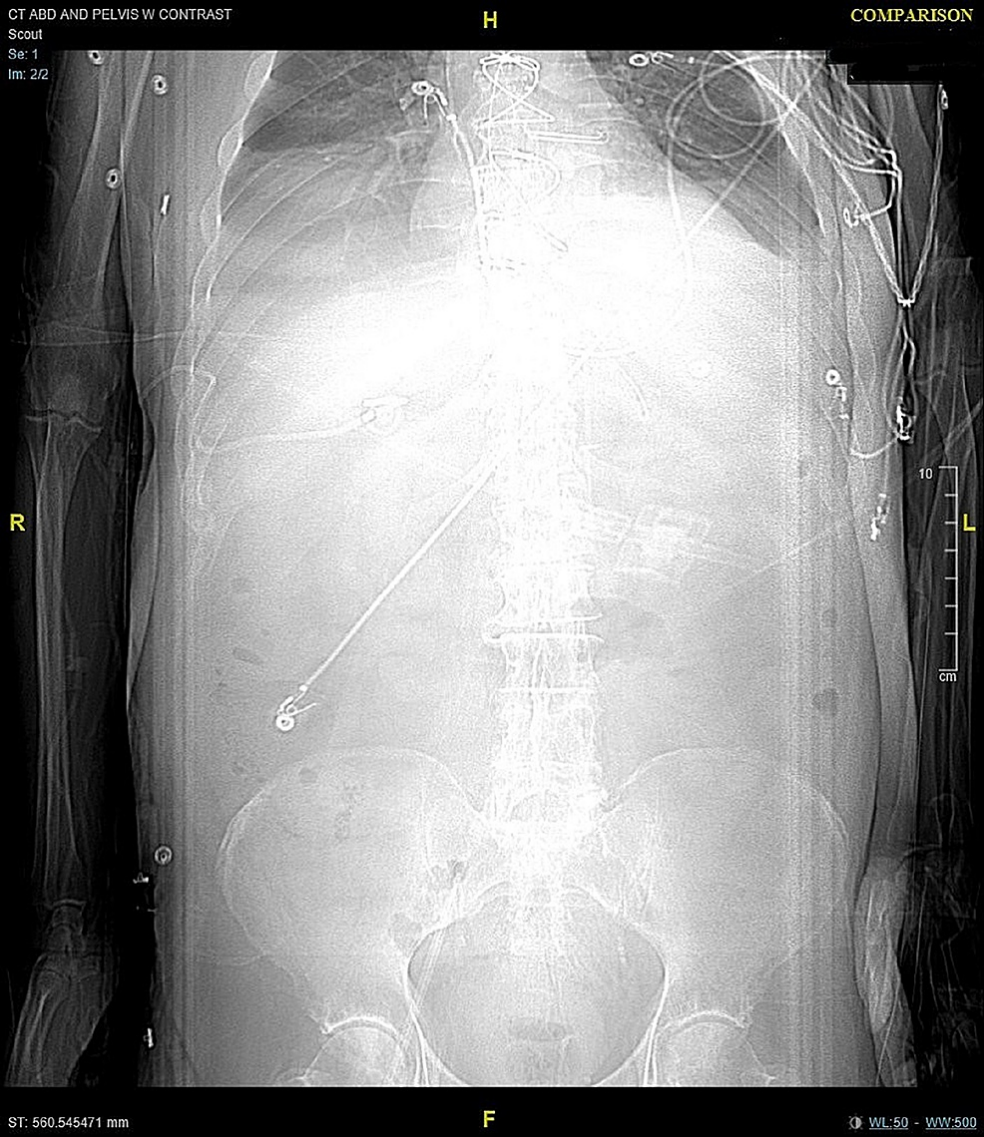
CT of the abdomen/pelvis on admission-scout. Scout: On admission, without abnormalities. CT: computed tomography

**Figure 2:**
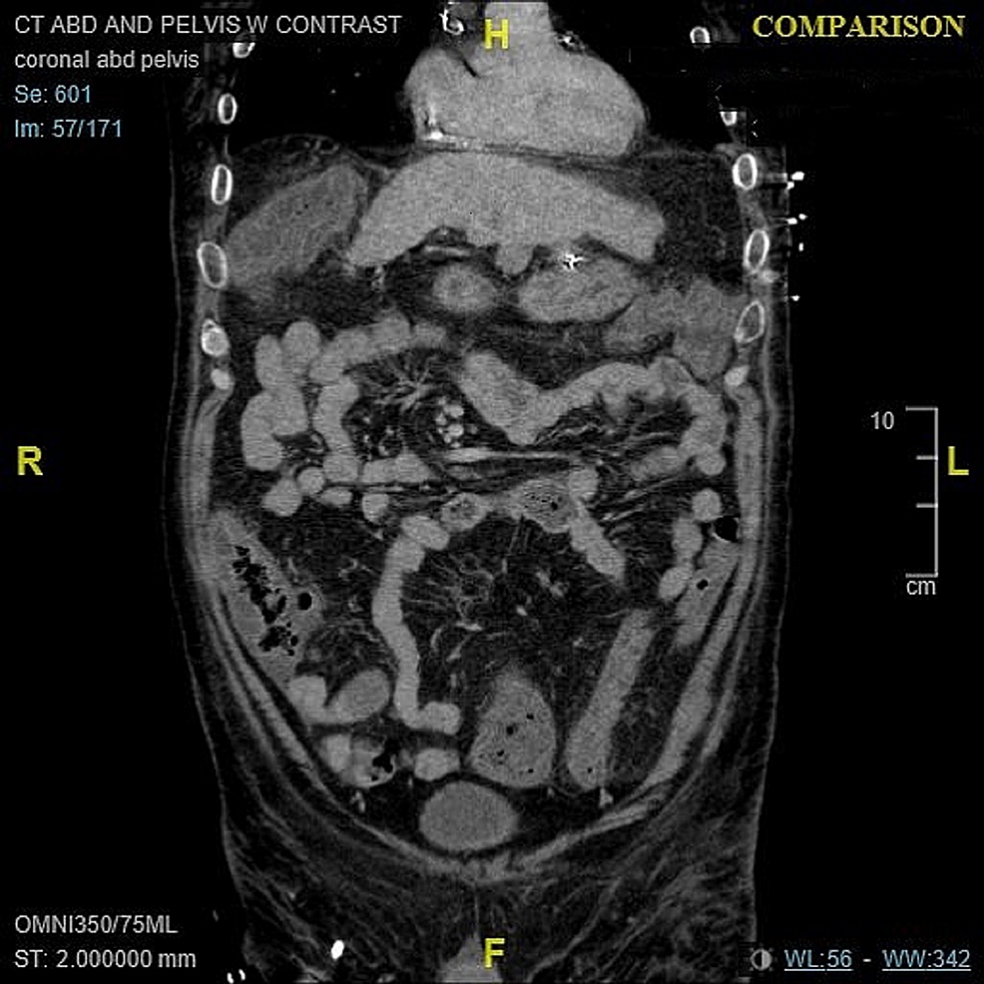
CT of the abdomen/pelvis on admission. Coronal view: On admission, marked diffuse colonic wall thickening with adjacent fat stranding, suggestive of colitis without dilated bowel (maximum right colon dilation of 4 cm), and nodular contour of the liver, suggestive of hepatic cirrhosis. CT: computed tomography

**Figure 3:**
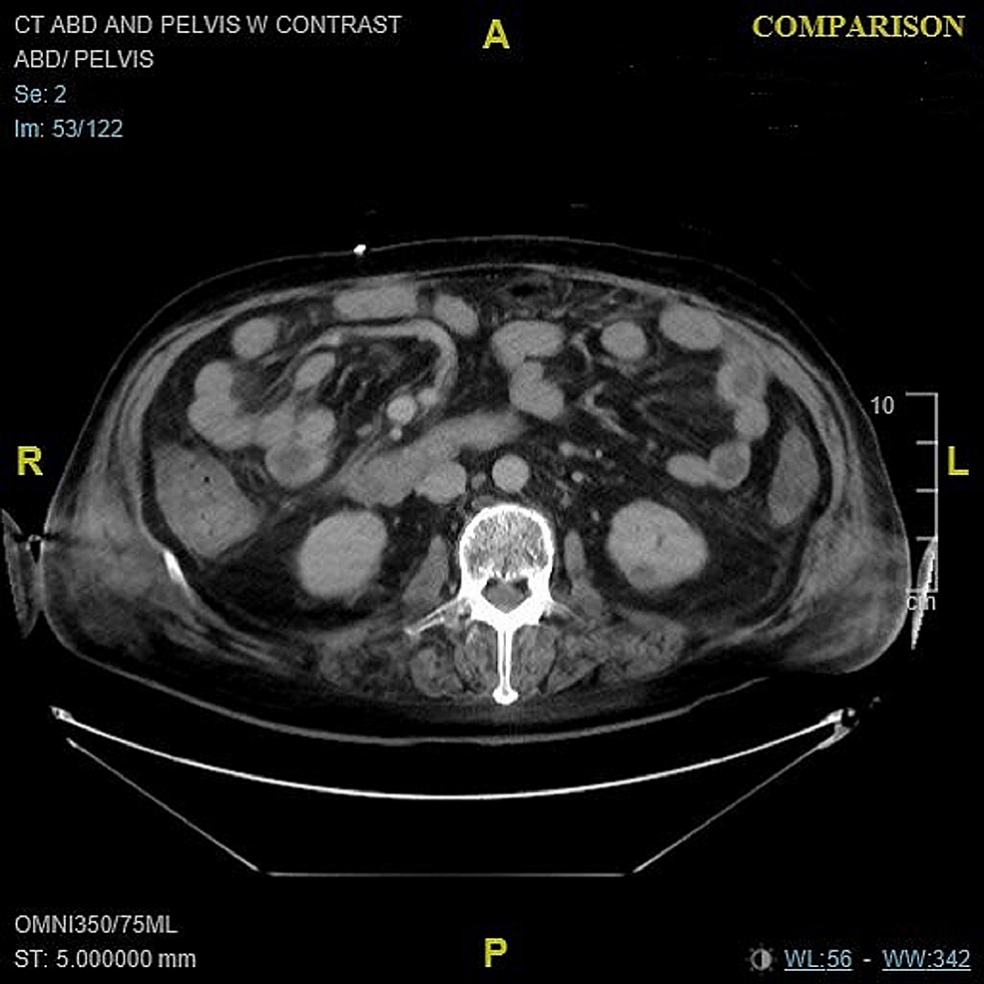
CT of the abdomen/pelvis on admission. Axial view: On admission, marked diffuse colonic wall thickening with adjacent fat stranding. CT: computed tomography

**Figure 4:**
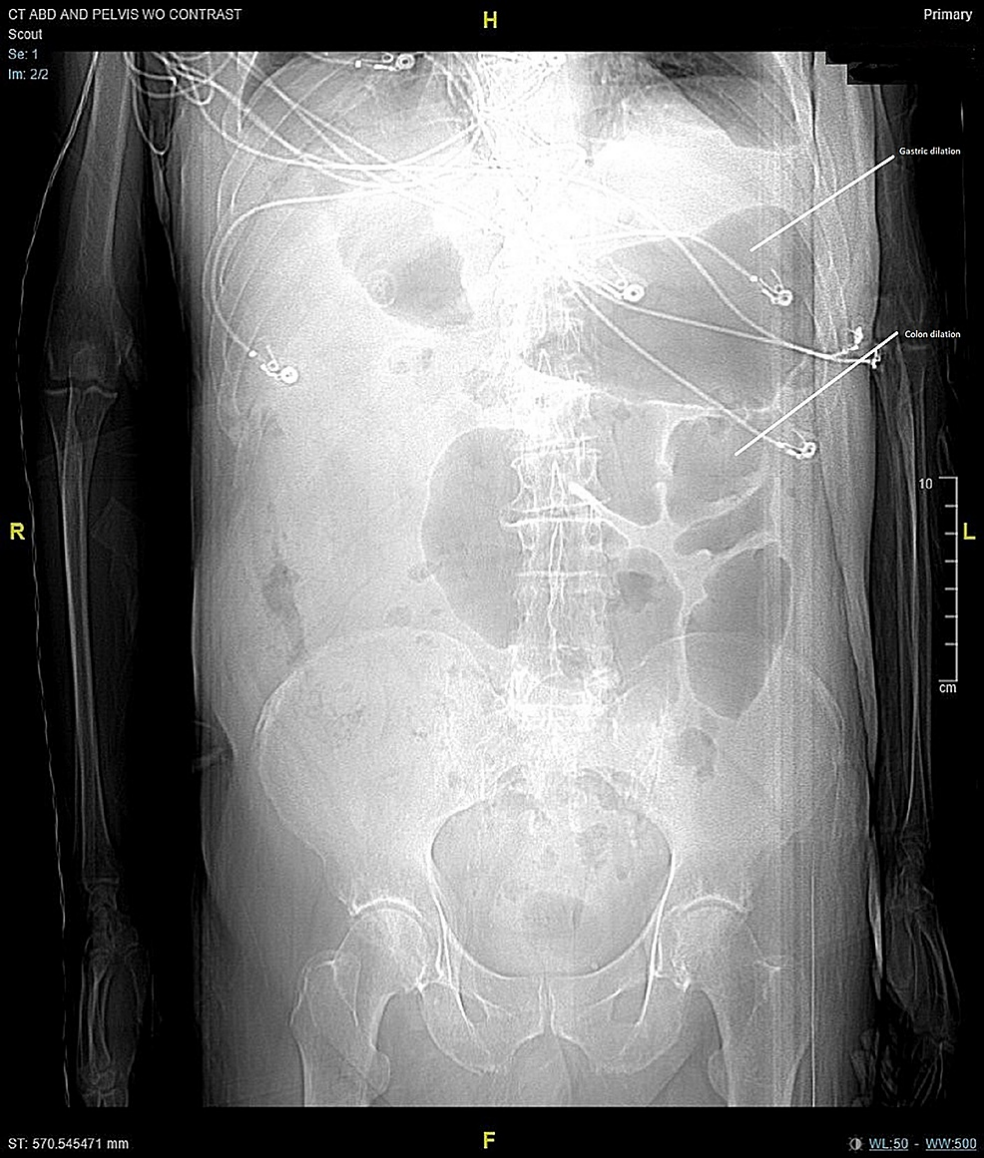
CT of the abdomen/pelvis on worsening of infection. Scout: Large bowel dilation. CT: computed tomography

**Figure 5:**
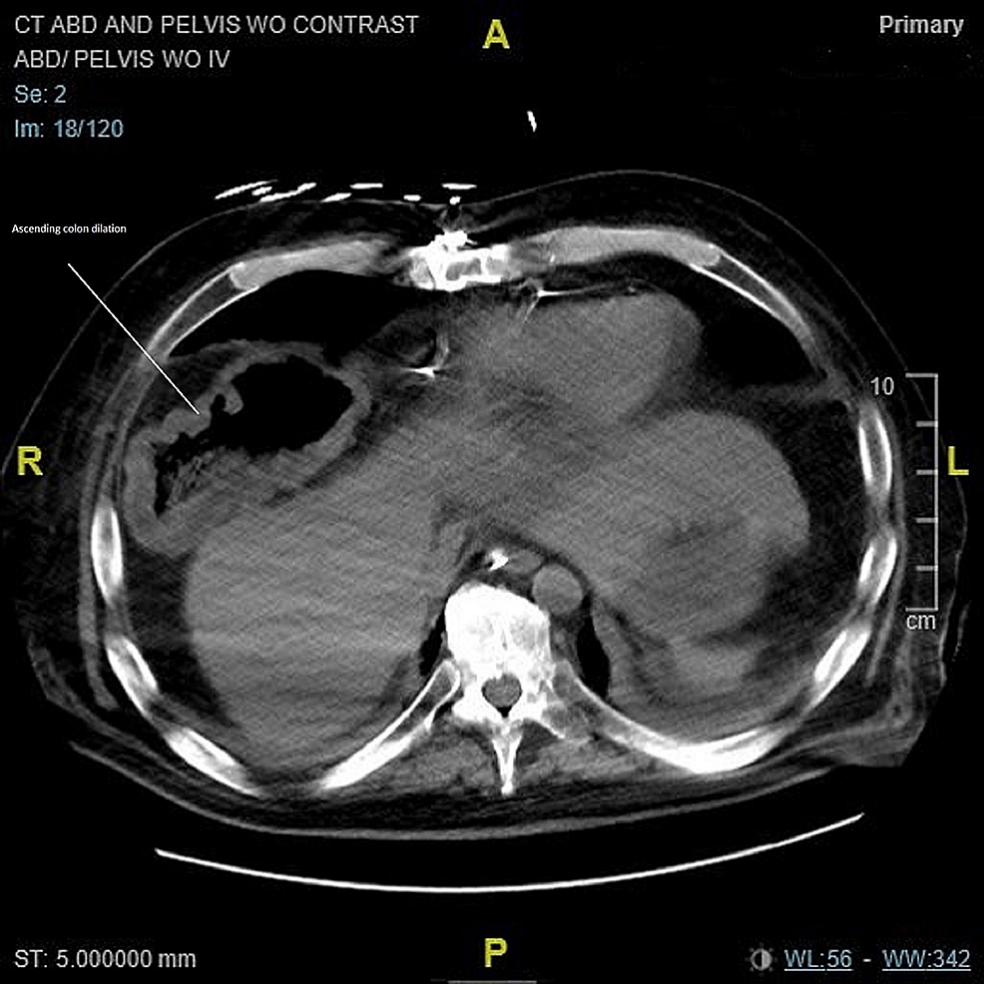
CT of the abdomen/pelvis on worsening of infection. Axial view: Circumferential thickening of the colon wall most marked within the right colon. Increased colonic wall thickening concerning for progression of infection. Before the use of fidaxomicin. CT: computed tomography

**Figure 6:**
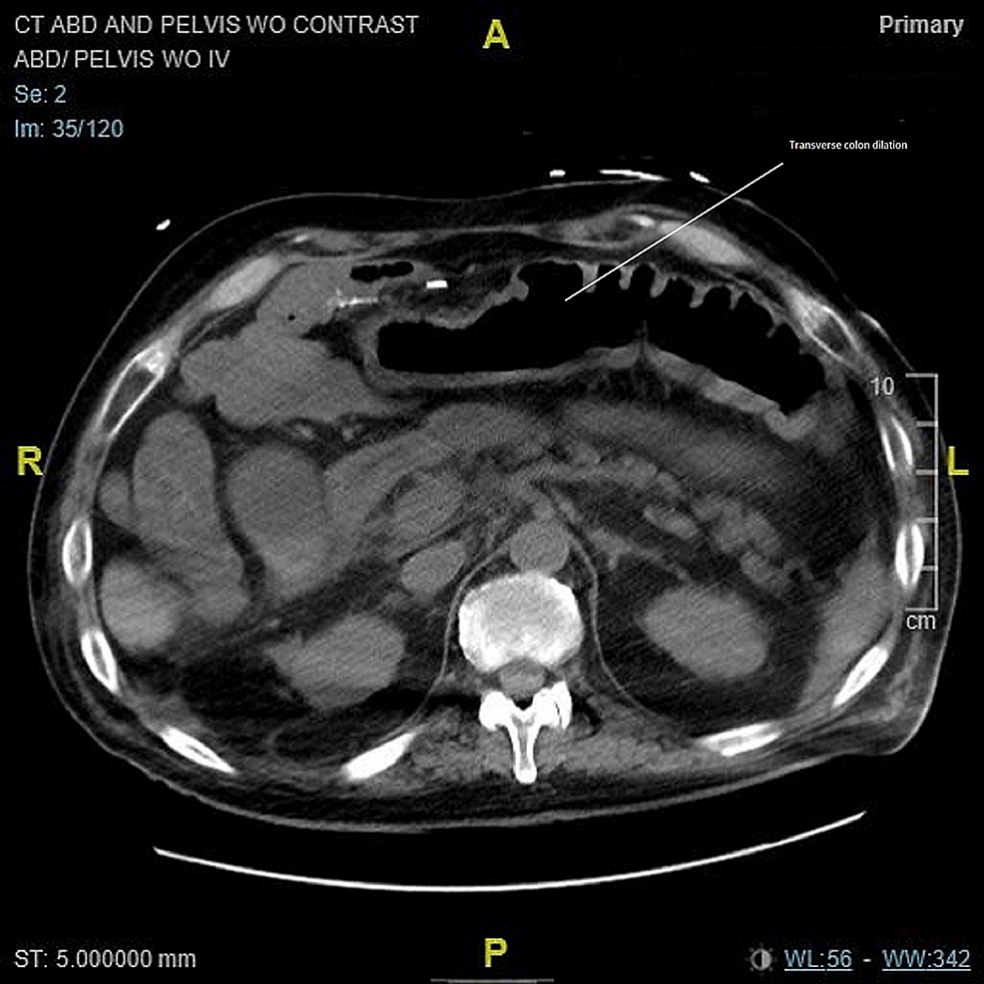
CT of the abdomen/pelvis on worsening of infection. Axial view: Circumferential thickening of the colon wall in the transverse colon. The small bowel is decompressed. Moderate upper abdominal ascites with a small amount of pelvic free fluid. Increased colonic wall thickening concerning for progression of infection. Before the use of fidaxomicin. CT: computed tomography

**Figure 7:**
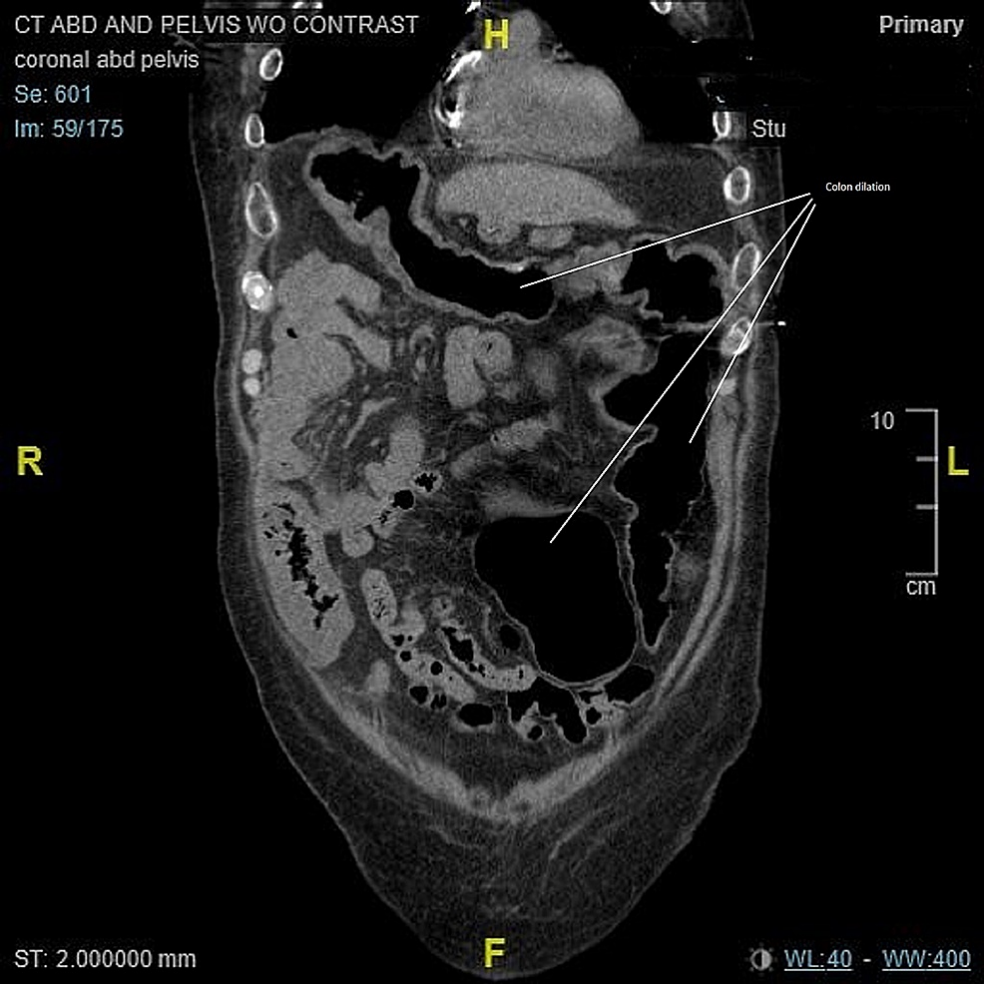
CT of the abdomen/pelvis on worsening of infection. Coronal view: Circumferential thickening of the colon wall most marked within the right colon and increased in the transverse colon. Increased colonic wall thickening concerning for progression of infection. Before the use of fidaxomicin. CT: computed tomography

In the ICU, his workup at admission showed pH 7.061, PCO_2_ 28 mmHg, PO_2_ 127 mmHg, HCO_3_ 7.9 mEq/L, creatinine 4.54 mg/dL, blood urea nitrogen 152 mg/dL, potassium 4.4 mEq/L, lactate 9.0 mmol/L, WBC 14.35 × 10^9^/L with a neutrophil count of 88%. *C. difficile* stool antigen and toxin genes were positive confirming the results of the external facility. Admission CT of the abdomen and pelvis showed a marked diffuse colonic wall thickening with adjacent fat stranding, suggestive of colitis without dilated bowel (maximum right colon dilation of 4 cm) and nodular contour of the liver, suggestive of hepatic cirrhosis (Figures 1-3). Upon arrival to our facility, the patient required immediate intubation and use of vasopressors; he was started on IV fluids, norepinephrine, and later on vasopressin. The antibiotic coverage for the septic shock was empiric use of IV vancomycin and piperacillin/tazobactam. CDI treatment was done with oral vancomycin 500 mg QID and IV metronidazole 500 mg TID. Nephrology was consulted for acute renal failure and the patient was dialyzed on the same day. General surgery was consulted; however, due his to critical condition and associated comorbidities, the patient was deemed to be a poor surgical candidate. Two days after admission to the ICU, the patient presented with worsening diarrhea with watery bloody stools. Another CT of the abdomen and pelvis (Figures 4-7) showed a worsening of colitis inflammation/infection besides an improvement in the WBC count (8.42 × 10^9^/L with a neutrophil count of 87.9%). Fidaxomicin was started, and within 24 hours of treatment, the patient had a rapid clinical stabilization with a reduction in diarrhea volume and a significant reduction in the use of vasopressors. The patient was extubated and transferred to the regular floor in four days. He completed a total of seven days of IV vancomycin and piperacillin/tazobactam and 15 days of IV metronidazole; fidaxomicin was continued for 10 days.

## Discussion

Oral vancomycin and parenteral metronidazole are the recommended therapy of choice for fulminant CDI according to the IDSA/SHEA 2017 guidelines. The strength of recommendation is considered to be strong based on moderate-quality evidence for both medications [5]. In our case, the patient was started on IV metronidazole and PO vancomycin following the guidelines but failed to improve, and his diarrhea and clinical conditions worsened. His CDI treatment was changed to fidaxomicin, but the use of IV vancomycin and piperacillin/tazobactam was continued due to concerns about superimposed bacterial infection. Although oral vancomycin and parenteral metronidazole are the treatment of choice for fulminant CDI, there is always a potential risk of intestinal microflora change and induction of drug-resistance bacteria, resulting in persistent infection and worsening the outcome.

Vancomycin is a bacteriostatic drug, whereas fidaxomicin is a bactericidal drug with an 18-membered-ring macrolytic antibiotic that inhibits RNA polymerase sigma subunit resulting in the inhibition of protein synthesis and bacterial death [8]. It produces a longer-lasting effect, expedites infectious recovery, and reduces recurrence. Fidaxomicin has a narrow-spectrum activity, and by sparing both gram-positive and gram-negative colonic microflora, it has very little effect on normal intestinal microbiota, maintaining a balanced competitive environment mediated by commensal bacteria, preventing *C. difficile* colonization, and potentially reducing drug resistance [9,10]. A study conducted in 2010 comparing the effect of fidaxomicin versus vancomycin on bowel microbiota in nonsevere disease demonstrated that patients treated with fidaxomicin had a 47% lower recurrence rate of CDI compared to vancomycin [11]. In addition to an initial recovery noted in patients treated with vancomycin, recurrence was more likely to occur due to its disruptive effect on normal intestinal microbiota, resulting in CDI spore germination and proliferation of vegetative cells [6,7]. Another study showing better outcomes with the use of fidaxomicin over vancomycin was reported among patients requiring the use of concurrent antibiotics due to severe infection superimposed to CDI. Usually, treatment guidelines recommend stopping all implicated antibiotics at the onset of CDI due to a lower cure rate (84.4% vs. 92.6%; P < 0.001) and an extended time to resolution of diarrhea (97 vs. 54 hours; P < 0.001) for patients who continue the use of antibiotics. The use of fidaxomicin was associated with an increased cure rate (90.0% vs. 79.4% P = 0.04) and fewer recurrences (16.9% vs. 29.2%; P < 0.048) compared to vancomycin [12-14].

Our patient had a poor prognosis due to his presentation with fulminant CDI and renal insufficiency according to a German retrospective study in patients with CDI and severe medical conditions. The resolution of diarrhea (67.5% and 68.0%) and mortality (24.6% and 27.6%) for patients with fulminant CDI and severe renal impairment were lower compared to 78% of diarrhea resolution and 30-day mortality rate of 17% for other severe medical conditions [9]. Our patient had a good clinical response to fidaxomicin besides the severe clinical condition of the patient that had failed to respond to the first-line antibiotic use with oral vancomycin and IV metronidazole. He had clinical stabilization with the resolution of diarrhea allowing transference from the ICU to the general floor.

## Conclusions

The use of fidaxomicin in CDI has been reported to be advantageous over vancomycin in different settings. Our case shows the successful use of fidaxomicin as a salvage treatment for a patient with fulminant CDI that failed to respond to the initial treatment with oral vancomycin and IV metronidazole. The impressive clinical improvement is encouraging; however, more studies are required to support the use of fidaxomicin as a salvage therapy for patients who fail to respond to first-line antibiotic treatment.
